# Significant traumatic atrophy of the spinal cord in connection with severe cervical vertebral body hypoplasia in a boy with Larsen syndrome: a case report and review of the literature

**DOI:** 10.4076/1757-1626-2-6729

**Published:** 2009-06-17

**Authors:** Ali Al Kaissi, Johannes Altenhuber, Franz Grill, Klaus Klaushofer

**Affiliations:** 1 Ludwig Boltzmann Institute of Osteology at the Hanusch Hospital of WGKK and, AUVA Trauma Centre Meidling, 4th Medical Department, Hanusch HospitalViennaAustria; 2 Orthopaedic Hospital of Speising, Paediatric DepartmentViennaAustria

## Abstract

**Introduction:**

Cervical kyphosis may be potentially the most serious and, indeed, a life-threatening manifestation of Larsen syndrome because of the impingement on the spinal cord at the apex of the kyphosis. Abnormalities of the spine, specifically cervicothoracic kyphosis requires specific attention and management.

**Case presentation:**

We report on a 3-year-old boy who presented with full clinical and the radiographic features of Larsen syndrome. There was significant vertebral body hypoplasia of C5/7 combined with spina bifida occulta from C1/T2, resulting in congenital cervical instability and kyphosis.

**Conclusion:**

Congenital or developmental cervical kyphosis is a serious orthopaedic abnormality, which is associated with several syndromic associations such as Larsen syndrome, diastrophic dysplasia, chondrodysplasia punctata, camptomelic dysplasia, and neurofibromatosis.

## Introduction

Abnormalities of the cervical spine, specifically cervical kyphosis, were not emphasised in the original description of Larsen syndrome [1]. Larsen et al., [1] called attention to a syndrome of multiple congenital dislocations associated with a characteristic facies. It is a rare, pathologic condition, characterized by multiple joint dislocations, distinctive deformities of the hands and feet, characteristic facial features (described as a “dish face,” with a saddle nose and hypertelorism), kyphoscoliosis, and segmentation anomalies of the vertebrae. Diverse treatment options, including conservative observation and surgical correction, have been reported for patients who present with cervical spine pathophysiology [1-4]. Many patients who have Larsen syndrome are described as being hypotonic, a feature that contributes to a delay in the achievement of motor skills, such as the ability to walk. Hyperlaxity and dislocations of joints are cardinal features and as an anterior dislocation of the knee is nearly always present, delay in walking and delay in other developmental milestones are mainly due to both hypotonia and orthopaedic problems [5-10]. Our patient manifested incomplete ossification in the posterior arches of the vertebral units associated with significant vertebral body hypoplasia of C5/7 resulting in congenital cervical instability and kyphosis. Severe atrophy of the spinal cord consistent with traumatic injury at C7 was the outcome. The purpose of this paper is to suggest that; cervical kyphosis may be potentially the most serious and, indeed, a life-threatening manifestation of Larsen syndrome because of the impingement on the spinal cord at the apex of the kyphosis. Abnormalities of the spine, specifically cervicothoracic kyphosis requires specific attention and management.

## Case presentation

We report on a 3-year-old boy of Austrian origin, referred to our orthopaedic department at the age of two months because of multiple deformities of the upper and lower extremities caused by multiple dislocations.

**Figure 1.**
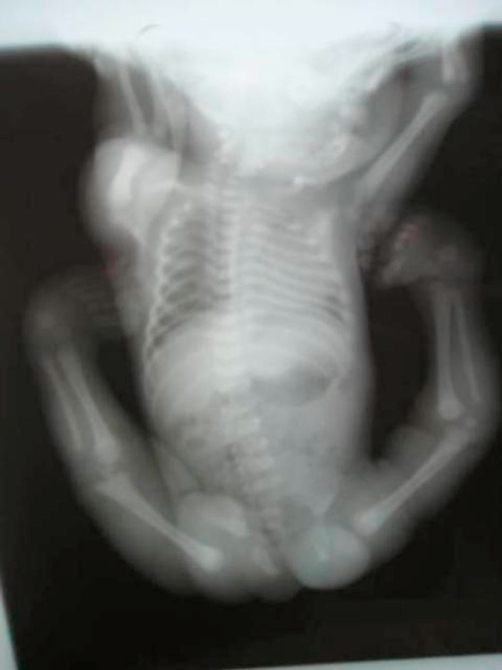
Anteroposterior whole skeleton radiograph at the age of one month showed multiple contractures associated with clubfoot, bilateral dislocation of elbows, hips and knees (most characteristically, anterior dislocation of the tibia on the femur), and short metacarpals with cylindrical fingers lacking the usual tapering were the prime skeletal features associated with undermineralization and overtubulation of the long bones were present.

**Figure 2.**
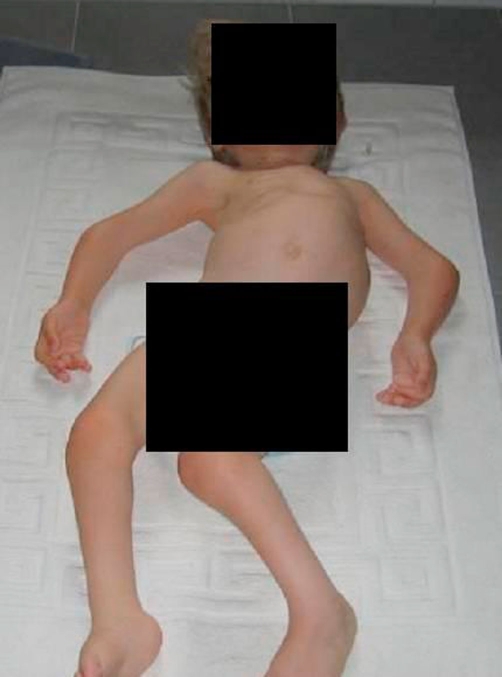
Floppiness, aggravated the situation resulted from trauma to the lowest cervical spine, and neurological examination was consistent with a severe spinal cord injury at C7.

**Figure 3.**
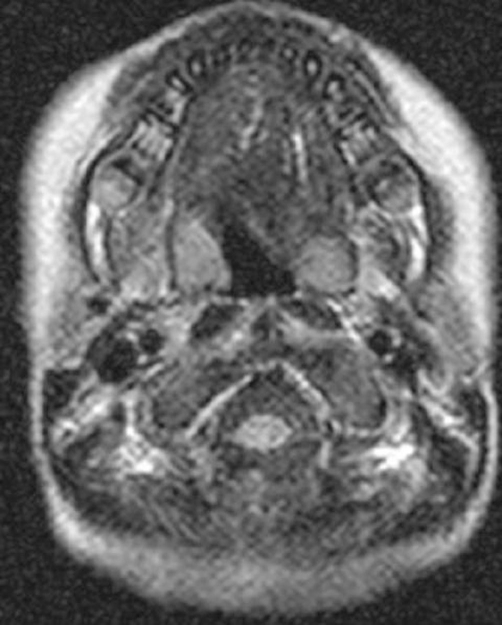
Axial MRI imaging of the craniocervical junction showed absence of the posterior arch of C1 (clefting).

**Figure 4.**
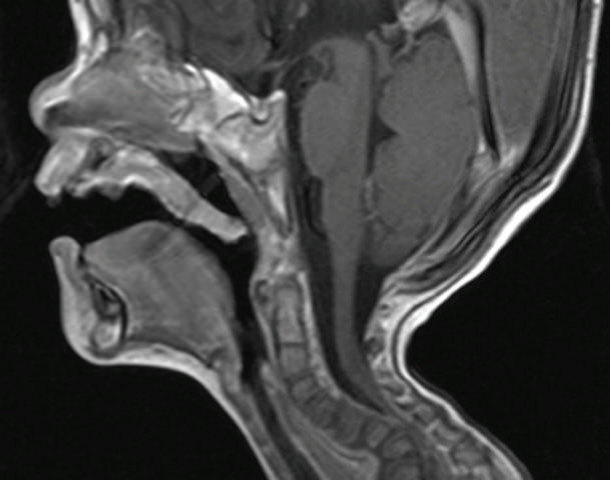
T2-weighted magnetic resonance imaging sequence that was made post intervention, showed severe impingement on the spinal cord at the level of C7. Dramatic progression of the kyphosis to 90 degrees had occurred. In addition significant hypoplasia of the cervical vertebral bodies along the spine segments C5/7.

**Figure 5.**
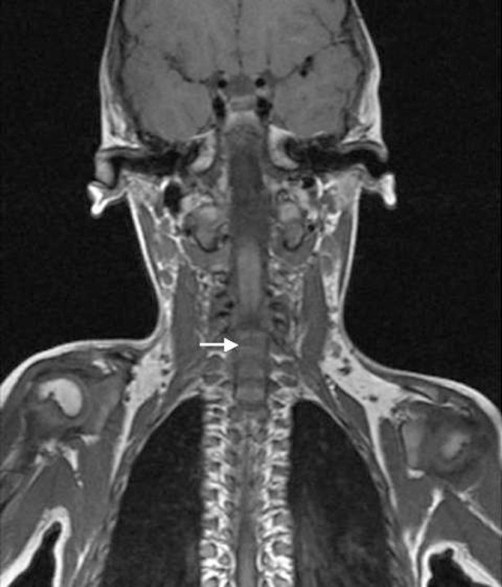
Coronal T2-MRI showed significant defective ossification of the vertebral bodies (arrow).

He was a product of 38 weeks gestation. At birth his growth parameters were -2SD. Parents were healthy and non-consanguineous, and the family history was unremarkable. At birth cleft palate associated with cervicothoracic kyphosis was identified. Multiple contractures associated with clubfoot, bilateral dislocation of elbows, hips and knees (most characteristically, anterior dislocation of the tibia on the femur), and short metacarpals with cylindrical fingers lacking the usual tapering were the prime skeletal features associated with undermineralization and overtubulation of the long bones were present) (Figure 1). Clinical features included a characteristic facies. Frontal bossing and the mid-face was hypoplastic with a depressed nasal bridge, and hypertelorism. At the age of four months he had been treated for the dislocated knees and clubfeet in long casts with a spreader bar and skeletal fixation pins above and below the knees. Repeated wedgings of the casts were carried out in an attempt to correct the feet. In parallel to this the child underwent closed reduction and he was fitted with a customized cervical orthosis. The kyphosis was partially corrected by extension. Floppiness, aggravated the situation resulted from trauma to the lowest cervical spine, and neurological examination was consistent with a severe spinal cord injury at C7 (Figure 2). Neuroimaging of the cervicothoracic spine showed a constellation of craniocervical anomalies. Axial MRI imaging of the craniocervical junction showed absence of the posterior arch of C1 (clefting) (Figure 3). T2-weighted magnetic resonance imaging sequence that was made post intervention, showed severe impingement on the spinal cord at the level of C7. Dramatic progression of the kyphosis to 90 degrees occurred. In addition, significant hypoplasia of the cervical vertebral bodies was present along the spinal segments of C5/7 (Figure 4). Coronal T2-MRI showed significant defective ossification of the vertebral bodies (Figure 5). All other investigations including abdominal ultrasound, echocardiodoppler, karyotyping and metabolic tests, which aimed to test calcium, phosphorus and vitamin D metabolism, were normal. Electromyography, peripheral nerve velocity studies, and a muscle biopsy were normal.

## Discussion

Larsen's syndrome is a rare inherited defect of connective tissue that is transmitted in both an autosomal dominant and recessive pattern. First described by Larsen [1], its cardinal findings consist of multiple congenital joint dislocations, usually of the hips, knees and elbows. Facially, there is frontal bossing, a depressed nasal bridge, hypertelorism and a flat facies. Deformities of the fingers (spatulate) and calcaneus (a double ossification centre), and spinal anomalies that may lead to major spinal instability and spinal cord injury, are important characteristics. Although abnormalities of the cervical spine were not emphasized in the original description of the syndrome [1], they may lead to serious complications.

Of the 9 affected infants followed by Johnston et al. [9], 5 were noted to have cervical kyphosis because of marked hypoplasia of 1 or 2 vertebral bodies (usually the fourth or fifth cervical vertebra, or both) at the apex of the kyphosis; the infants were successfully managed by posterior cervical arthrodesis alone. Johnston et al. [9], suggested that the prevalence of cervical kyphosis in Larsen syndrome has probably been underestimated, but may easily be documented because no dynamic studies or cooperation by the patients are necessary.

Laville et al. [2], reviewed thirty-eight patients with Larsen syndrome who came from an isolated geographical area and did not find any who had cervical kyphosis. In that series, however, the absence of the accessory calcaneal apophysis, which is a characteristic radiographic finding in classic Larsen syndrome, casts doubt on the diagnosis and, hence, on the validity of the failure to observe cervical deformity. It might however just have been underdiagnosed. The potential morbidity and mortality from this deformity are obvious. Indeed, one of the patients of Micheli et al. [7], died at the age of twenty-six months as a result of compression of the spinal cord, before the deformity of the neck could be treated. Also, one of the original patients of Larsen et al, [1,2] died at the age of one year after open reduction of a dislocation of the knee. The death was attributed to so-called respiratory anaesthetic complications.

Diverse treatment options in patients with Larsen syndrome, including conservative observation and surgical correction, have been reported for patients who present with cervical spine pathophysiology. Differences in surgical approaches, timing of the correction, and pre- or postoperative bracing have been reported [10-12].

Lachman [13] described the poor ossification and or developmental failure of the cervical vertebrae has been described exclusively in patients with skeletal dysplasias. Lethal and non-lethal types have been described such as, campomelic dysplasia, diastrophic dysplasia, chondrodysplasia punctata, opsismodysplasia, Desbuquois dysplasia, achondrogenesis type IA and IB and achondrogenesis type II and others.

## Conclusion

We wish to stress that the development of a severe rigid cervico-thoracic kyphosis or neurological deficits, or both. Posterior arthrodesis for cervical kyphosis should take precedence in the staging of the multiple orthopaedic procedures that are necessary for a patient who has Larsen syndrome.

## Abbreviations

MRI: Magnetic resonance imaging
SD: Standard deviation
